# Prioritizing Pregnant Women for Long-Lasting Insecticide Treated Nets through Antenatal Care Clinics

**DOI:** 10.1371/journal.pmed.1001717

**Published:** 2014-09-09

**Authors:** Jenny Hill, Jenna Hoyt, Anna Maria van Eijk, Feiko O. ter Kuile, Jayne Webster, Richard W. Steketee

**Affiliations:** 1 Department of Clinical Sciences, Liverpool School of Tropical Medicine, Liverpool, United Kingdom; 2 Disease Control Department, London School of Hygiene & Tropical Medicine, London, United Kingdom; 3 Malaria Control and Elimination Program, PATH, Seattle, Washington, United States of America

## Abstract

Jenny Hill and colleagues discuss the importance of antenatal care services in providing pregnant women with a long-lasting insecticide treated net for the prevention of malaria in both the mother and infant.

*Please see later in the article for the Editors' Summary*

**Summary Points**

- Long-lasting insecticide treated nets (LLINs) are a powerful public health tool and, when used by pregnant women, contribute to improving maternal, neonatal, and infant health, with lasting benefits to the developing child.
- Use of LLINs among pregnant women is well below national and international targets; the median use of an insecticide treated net (ITN) the previous night among pregnant women across 37 countries for 2009–2011 was 35.3% (range, 5.2%–75.5%); ITN use was higher in areas with both a high disbursement of funds for malaria control and a lower per-head gross domestic product.
- Routine antenatal care (ANC) services constitute an important delivery channel that ensures pregnant women who attend an ANC clinic at least once (77% in sub-Saharan Africa) are covered with a LLIN from their first ANC visit in each pregnancy and plays an important role in maintaining population-level coverage between campaigns, particularly for women who become pregnant between campaigns and for infants born outside of campaign years.
- The majority of LLINs delivered from 2010–2012 in sub-Saharan Africa were through mass campaigns as countries sought to reach the 80% coverage target, and some of the LLINs used in these campaigns were re-allocated from routine ANC delivery.
- Going forward, national malaria programmes and donors alike will have to make difficult decisions to balance costs with the benefits and impact of investments in LLINs. Where choices must be made, high-risk groups (pregnant women and children under 5 years of age) should be prioritized for the same reason these groups were targeted under the pre-universal coverage WHO strategy.

The use of insecticide treated nets (ITNs), and subsequently the new generation of long-lasting insecticide treated nets (LLINs), has been a core malaria prevention strategy for more than two decades [1], and until 2010, distribution of LLINs targeted biologically vulnerable groups such as pregnant women and children aged less than 5 years [2],[3]. In 2008, due largely to increased funding for malaria control leading to impressive gains in LLIN coverage, the Roll Back Malaria (RBM) Partnership set a more ambitious target of universal coverage of LLINs, defined as universal access to, and use of, LLINs [4],[5].

The strategy for achieving and maintaining universal coverage outlined by the RBM Partnership involves a combination of strategies based on mass campaigns, either target-specific or population-wide, to rapidly scale up coverage (“catch up”), complemented by continuous distribution through routine health services, including antenatal clinics, child health clinics, and expanded programme on immunisation (EPI) services (“keep up”) [6]. The choice of the combination is generally based on existing coverage and status of available distribution mechanisms in a given country. It is well recognised that, individually, each mechanism is suboptimal to maintain universal coverage and will leave some gaps.

Use of ITNs among pregnant women is well below national and international targets; a recent meta-analysis of national survey data in 37 countries for the years 2009–2011 estimated the median use of an ITN the previous night among pregnant women was 35.3% (range 5.2%–75.5%) [7]. ITN use was higher in areas with both a high disbursement of funds for malaria control and a lower per-head gross domestic product. Younger or adolescent, unmarried, and less educated women are significantly less likely to use ITNs, which may be related to lower affordability and in-household access among these women [8].

## Public Health Rationale for Net Distribution to Pregnant Women

LLINs are a powerful public health tool and, when used by pregnant women, contribute to improving maternal, neonatal, and infant health, with long-lasting benefits to the developing child. Worldwide, an estimated 125 million pregnancies are at risk from malaria each year [9]. Pregnant women are 1.5 times more susceptible to malaria infection than non-pregnant women [10] and malaria infection can have devastating consequences on maternal, newborn, infant, and child health. In Africa, 10,000 women [11],[12] and between 75,000 and 200,000 infants [13],[14] are estimated to die annually as a result of malaria infection during pregnancy, and approximately 11% (100,000) of neonatal deaths are due to low birth weight (LBW) resulting from *Plasmodium falciparum* infections in pregnancy [15]. In the absence of malaria control in pregnancy, it is estimated that 11.4 million (95% credible interval [CrI], 10.7–12.1) pregnancies would have experienced *P. falciparum* placental infection at some stage of pregnancy, accounting for 41% of the estimated 27.6 million live births in sub-Saharan Africa in 2010 [16]. Combined with estimates of the relationship between placental infection and the risk of LBW, 900,000 (95% CrI, 530,000–1,240,000) LBW deliveries per year were estimated to be caused by placental malaria. The end of the first trimester is a key period during which 65% (95% CrI, 61%–70%) of the potentially infected pregnancies first experience infection, and primigravidae experience a high proportion 39% (95% CrI, 33%–46%) of the total potential malaria-attributable LBW burden.

LLINs have been proven in clinical trials and in field programs to substantially reduce the adverse consequences of malaria in pregnancy, reducing maternal anaemia, severe anaemia, peripheral and placental malaria, and low birth weight [17]–[19], and LLINs are highly cost effective [20]. As a consequence, LLINs, along with intermittent preventive treatment in pregnancy (IPTp) [17],[21],[22], together with effective case management of malaria, are recommended by WHO in malaria endemic settings in Africa. At 2012 coverage levels across 32 countries in sub-Saharan Africa, LLIN or IPTp use among women in their first or second pregnancies was significantly associated with a decreased risk of neonatal mortality (incidence rate ratio 0·82; 95% confidence interval (CI), 0·698–0·96) and reduced odds of low birth weight (adjusted odds ratio 0·79; 95% CI 0·73–0·86), compared with newborn babies of mothers with no protection, after controlling for potential confounding factors [23].

## Routine Distribution through Antenatal Care Clinics—An Important “Keep Up” Strategy

The delivery of free or subsidized LLINs (or vouchers) to pregnant women through ANC services is a key strategy for controlling malaria and increases coverage and use by both pregnant women [24]–[27] and their infants [24],[25]. As infants in most malaria-endemic settings sleep with their mother during the first year of life (or longer), the protective effect of an LLIN delivered to a pregnant woman is therefore extended through the infant's first year of life.

Routine ANC services constitute an important delivery channel that ensures pregnant women who attend ANC at least once (77% in sub-Saharan Africa) [28] are covered with an LLIN from their first ANC visit and in subsequent pregnancies and plays an important role in maintaining population-level coverage between campaigns, particularly for women who become pregnant between campaigns and for infants born outside of campaign years [29],[30]. Whilst mass campaigns can rapidly scale up coverage, by as much as 30%–80% [31], universal coverage will not be maintained without the continuous distribution of LLINs, and ANC routine services have proven effective for reaching pregnant women [28],[32]–[35].

In addition, campaign delivery of LLINs to households with pregnant women [36], households with children under 5 years of age [37], or households with low socioeconomic status [38] has shown limited impact on increasing coverage among pregnant women [8], supporting the need for routine ANC services. Notwithstanding important limitations of modelling studies, which in the absence of evidence use some assumptions (costs, efficiencies of scale, data from a limited number of countries, etc.), modelling has demonstrated that a combination of an ANC- and school-based distribution would sustain the high coverage achieved in recent years by the mass campaigns [39]. Modelling also predicts that supplementing mass distribution campaigns with ANC delivery could achieve a 1.4 times greater reduction in child mortality than mass distribution alone, as children born between campaign years would be covered during the most vulnerable time [40]. Delivery of LLINs through ANC to pregnant women is an effective, sustainable strategy for continuous distribution [41]; greater effort is needed to encourage women to initiate ANC attendance early in the first trimester, and promoting the availability of a free ITN at early ANC booking may encourage women to initiate ANC earlier [41].

In short, the distribution of LLINs through routine services, ANC services included, is an important strategy and will require a sustained commitment to health systems strengthening; and neglecting this strategy will impede a country's ability to maintain universal coverage over the longer term. Delivery of LLINs through ANC has been observed to increase pregnant women's attendance at ANC clinics [42], which is an important platform through which women receive other essential antenatal care services, such as prevention of mother to child transmission of HIV (PMTCT); management of anaemia, syphilis, and other conditions; birth planning; etc. In addition, ANC clinics provide an opportunity to educate, inform, and encourage women to use ITNs.

## Recent Policy and Funding for LLINs among Key Donors and Partners

The policy shift towards universal coverage reflects huge progress in malaria control and is a laudable goal that has injected enthusiasm into the global malaria community and has attracted calls for elimination. Notwithstanding, funding for malaria control peaked at $US2 billion in 2011 [43] and has begun to decline, ushering in an era of limited resources. Amidst the push to achieve universal coverage and dwindling resources, there is the potential danger whereby “keep-up” strategies lose resources and funding to its more attractive “catch-up” counterpart.

Despite recent encouraging statistics on funding for continuous delivery systems, including ANC, increasing from 22% in 2008–2010 of all funding commitments to 42% for the 2012–2016 funding interval [44], the funding gap has meant that the routine systems are the first to be left unfunded. One estimate for 2013–2016 suggests current funding commitments meet just over half of countries' needs, leaving a funding gap of approximately 374 million LLINs [43], and in a funding review of the Global Fund to fight AIDS, Tuberculosis, and Malaria and other major donors the authors report that 70% of as-yet-unfunded LLINs are for continuous delivery systems [44]. The majority of LLINs delivered from 2010–2012 in sub-Saharan Africa were through mass campaigns as countries sought to reach the 80% coverage target [6],[43]. Some of the LLINs used in these campaigns were re-allocated by national planners from routine ANC delivery to fill gaps in campaigns, as reported in Angola (2013), Cote d'Ivoire (2008), Cameroon (2011), Democratic Republic of Congo (2012, 2013), Kenya (2011), Malawi (2011), Nigeria (2014), Togo (2011), and Uganda (2012, 2014) (Matthew Lynch, Johns Hopkins University, personal communication, June 2014).

These trends prompted a policy recommendation from the WHO Vector Control Technical Expert Group to the Malaria Policy Advisory Committee (MPAC) noting that, although universal coverage was still the priority, LLINs distributed through routine channels such as ANC and EPI should continue regardless of mass campaign timing, and that nets for routine distribution should not be diverted to campaigns. This recommendation has been approved by MPAC [45] and a policy recommendation published [5].

## Recommendations

The shortfall in funding for malaria, generally, and for LLINs, in particular, calls for endemic country programs, malaria donors, implementing agencies, and partners to adopt the most cost-effective strategies to deliver this life-saving intervention. The challenge will be to ensure that population-wide coverage does not fall while maintaining highest priority for pregnant women and children. The arguments for maintaining the ANC distribution mechanism are strong. This mechanism reaches the highest risk population of mothers and their newborns, takes advantage of the fact that most pregnant women visit ANC clinics, is the only antenatal malaria prevention intervention that provides protection in the first trimester of pregnancy, and adds an important benefit to the focused ANC delivery system as it serves to encourage ANC attendance.

Going forward, national malaria programmes and donors alike will have to make difficult decisions to balance costs with the benefits and impact of investments in LLINs. WHO's MPAC has recommended that routine LLIN distribution (through ANC and the EPI) continue “before, during, and after” campaigns, and that recommendation needs to be adopted by Ministries of Health and donors [5],[45]. For routine distribution to continue, unaffected by campaigns, donors need to make their funding commitments for LLIN procurement for both routine and campaign delivery explicit and well in advance (2 years minimum), to allow governments to plan ahead for both catch-up and keep-up. Governments will need to track both stock of LLINs and their coverage and ensure that there are sufficient commodities for delivery through both routine and campaign strategies, requiring quality data on ANC delivery of LLINs, both through strengthened Health Management Information System reporting of LLIN distribution and through national surveys. Where choices must be made, high-risk groups (pregnant women and children under 5 years of age) should be prioritized for the same reason these groups were targeted under the pre-universal coverage WHO strategy. Receiving a net as an integral part of antenatal care sends a powerful message to a pregnant woman that this tool is important to protect herself and her child. Ministries of Health need to maximise ANC opportunities, for example, to use LLINs delivery at ANC clinics to promote earlier and increased demand for ANC, and vice versa.

## Abbreviations

ANC: antenatal care
CI: confidence interval
CrI: credible interval
EPI: expanded programme on immunisation
IPTp: intermittent preventive treatment in pregnancy
ITN: insecticide treated net
LBW: low birth weight
LLIN: long-lasting insecticide treated net
MPAC: Malaria Policy Advisory Committee
PMTCT: prevention of mother to child transmission of HIV
RBM: Roll Back Malaria
